# Filter-Based Phase Shifts Distort Neuronal Timing Information

**DOI:** 10.1523/ENEURO.0261-17.2018

**Published:** 2018-04-19

**Authors:** Dorin Yael, Jacob J. Vecht, Izhar Bar-Gad

**Affiliations:** 1 The Leslie and Susan Goldschmied (Gonda) Multidisciplinary Brain Research Center, Bar-Ilan University, 52900, Ramat-Gan, Israel; 2 Deuteron Technologies, 9777403, Jerusalem, Israel

**Keywords:** filters, neurophysiology, oscillations, phase, timing, waveform

## Abstract

Filters are widely used for the modulation, typically attenuation, of amplitudes of different frequencies within neurophysiological signals. Filters, however, also induce changes in the phases of different frequencies whose amplitude is unmodulated. These phase shifts cause time lags in the filtered signals, leading to a disruption of the timing information between different frequencies within the same signal and between different signals. The emerging time lags can be either constant in the case of linear phase (LP) filters or vary as a function of the frequency in the more common case of non-LP (NLP) filters. Since filters are used ubiquitously online in the early stages of data acquisition, the vast majority of neurophysiological signals thus suffer from distortion of the timing information even prior to their sampling. This distortion is often exacerbated by further multiple offline filtering stages of the sampled signal. The distortion of timing information may cause misinterpretation of the results and lead to erroneous conclusions. Here we present a variety of typical examples of filter-induced phase distortions and discuss the evaluation and restoration of the timing information underlying the original signal.

## Visual Abstract

**Figure:**
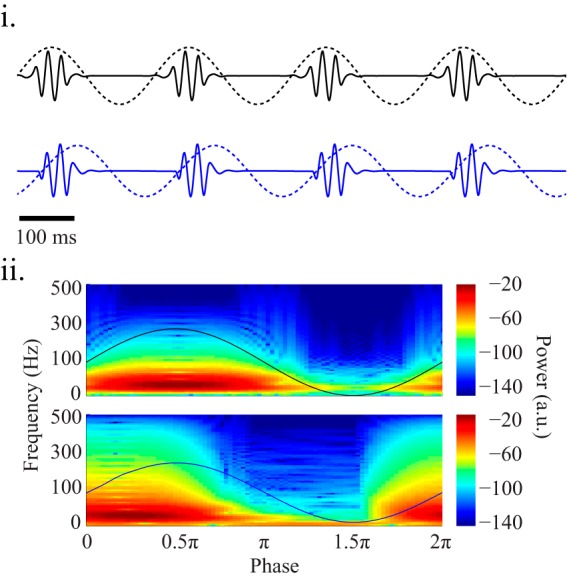

## Significance Statement

Filters are a common tool used in the processing of neuronal signals. In addition to their effect on the amplitude of different frequencies, filters also have a significant impact on their phases, which results in the distortion of the underlying timing information. This distortion, which arises by the online filters used in most neurophysiological systems and is exacerbated by further offline filtering, may cause severe misinterpretation of the results and lead to false conclusions. This manuscript presents different cases in which the timing information is disrupted and discusses the evaluation and correction of the underlying phase shifts.

## Introduction

**Figure 1.**
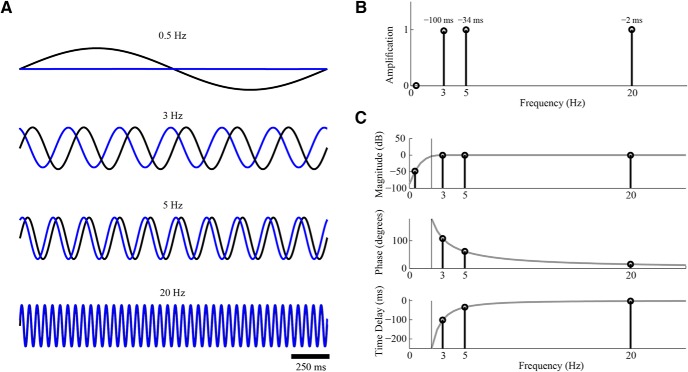
Filter induced magnitude and phase changes in the signal. The changes induced by a 2-Hz high-pass four-pole Butterworth filter. ***A***, Differential effect on four sinusoidal signals (black –raw signal and blue-filtered signal). ***B***, The amplification and phase change in the signals following the filtering. ***C***, The amplitude (top), phase (middle), and temporal (bottom) responses of the filter over all frequencies.

Filters are one of the most commonly used signal processing tools in neuroscience. Different types of filters are used in multiple applications ranging from online to offline, from analog to digital and from hardware to software in their implementation. These filters are applied to neurophysiological signals on different temporal and spatial scales as well as supplementary signals such as sensory stimuli or motor activity. The typical perceived role of these filters is to attenuate certain frequencies or frequency bands from the original signal. As a result, most neuroscientists focus on the magnitude of the modulation of the different frequencies; e.g., a certain high pass filter may reduce the magnitude of oscillations below 1 Hz within the original signal by 20 dB. Filters, however, do not only change the magnitude of the oscillations but also their phase, resulting in a temporal displacement. Some filters, termed linear phase (LP) filters, cause a fixed change in the temporal shift of all the frequencies. However, most filters, termed non-LP (NLP) filters, cause a differential time shift as a function of frequency (Oppenheim and Schafer, 1975; Oppenheim et al., 1999). A full description of the filter effect on the signal should thus comprise of the changes to both the magnitude and the phase of oscillations at different frequencies. The output signal of the filter follows a transformation in which some oscillations are reduced, other oscillations are not reduced but rather shifted in time, and still others are unchanged (or minimally altered) in either magnitude or phase (Fig. 1). Changes in oscillation phases lead to complex changes in the timing of oscillatory events, the distortion of the temporal relationship between oscillations at different frequencies and in different signals and alterations in the multi-frequency composition of the signal. These unexpected changes can lead to misinterpretation of the results and potentially introduce erroneous conclusions regarding the neuronal processes underlying the observed dataset. This manuscript presents common examples of these temporal distortions, generalizes the phenomena underlying each example, and finally suggests ways to address and correct these distortions.

## Filter-Induced Displacement of Phase and Time

The raw neurophysiological signal contains, in many cases, high energy in the low frequencies which may lead to saturations during subsequent sampling. This issue is addressed in most systems by online, hardware based, high-pass filters which attenuate these very low frequencies. The cutoff value of this filter varies dramatically and typically depends on the oscillations of interest to the researcher: a study of 0.5-Hz oscillations, for example in epilepsy (Vanhatalo et al., 2004), might use a 0.1-Hz high-pass filter whereas a study of 5-Hz oscillation, for example in Parkinson’s disease, might use a 1-Hz high-pass filter (Ben-Pazi et al., 2001). Once the data are acquired, scientists tend to overlook this initial filter and consider its output, often termed the wide-band pass filtered signal, as the equivalent of the raw analog electrophysiological signal, except for the attenuated frequencies. However, different components within this signal are actually shifted in time relative to the raw signal. In the best case, the time shift is constant for all frequencies (LP filter) which leads to a change in the perceived timing of the neurophysiological data relative to its original timing. However, in the more common case (NLP filter), the time shift varies for different frequencies, with those closest to the cutoff frequency typically being offset by the largest temporal change. For high-pass filters, the phases of frequencies near the cutoff frequency lead the phase of the raw signal whereas frequencies further away from the cutoff frequency have smaller shifts (Fig. 1). This results in a situation in which the relative phase (or time) shift between two oscillations at different frequencies is distorted, disrupting the internal composition of the signal. This may introduce an erroneous interpretation of the phase relation and assumptions as to which activity chronologically leads, and potentially causes or functionally leads the other activity.

**Figure 2.**
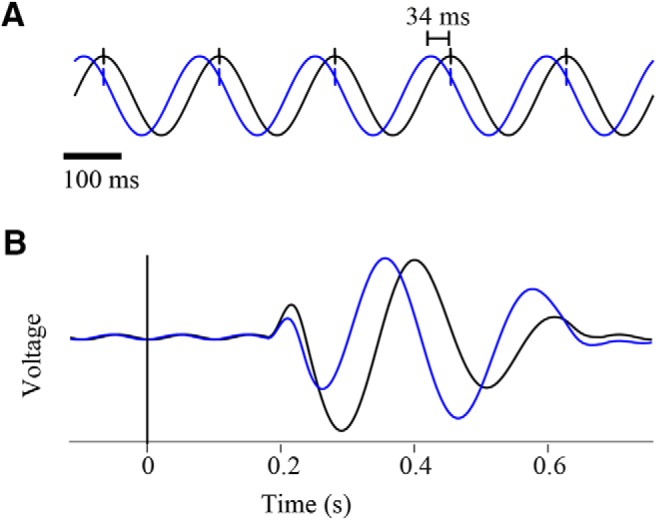
Filter-induced phase shifts of low frequencies. ***A***, Differential effect of filtering on the phase of the LFP (5 Hz) and action potentials (1000 Hz; cutoff frequency: 2 Hz). ***B***, Filter induced phase shifts leading to changes the timing and wave form of the filtered signal in relation to an external event (cutoff frequency: 2 Hz). Black-raw signal and blue–filtered signal.

A significant disruption of the internal order and temporal relationship within the same signal occurs when the signal is comprised of different frequencies, specifically when some of the prominent frequencies are close to the cutoff frequency of the filter resulting in a significant phase shift, while the others are distant resulting in a minor phase change. A typical example of this scenario is an extracellularly recorded signal containing both high frequency spikes and low frequency local field potentials (LFPs; Moran and Bar-Gad, 2010). Extracellular action potentials (spikes) consist of frequencies around 1 kHz, whereas the LFP signal contains low (starting from sub-Hz) frequencies. The low frequencies in the LFP signal are shifted in the filtered signal, appearing tens of milliseconds before their “real” time in the raw signal and relative to spikes whose timing is (almost) unaltered (Fig. 2*A*).

A similar disruption of the temporal relationship between two signals can occur in studies examining the interaction between an external event and the neuronal activity. The neuronal signal is aligned to an external event and averaged around it, thus enabling researchers to explore questions dealing with the magnitude and timing of responses of the targeted neuronal systems to external events, such as sensory stimuli. However, while the timing of the external event is fixed, the timing of the recorded signal is altered because of the phase shift, resulting in a disrupted temporal relationship between the two (Fig. 2*B*). The response times of neuronal activity or the exact timing of different components [i.e., the N400 visible within the event related potential (ERP)] within the signal may shift (Kutas and Federmeier, 2011).

**Figure 3.**
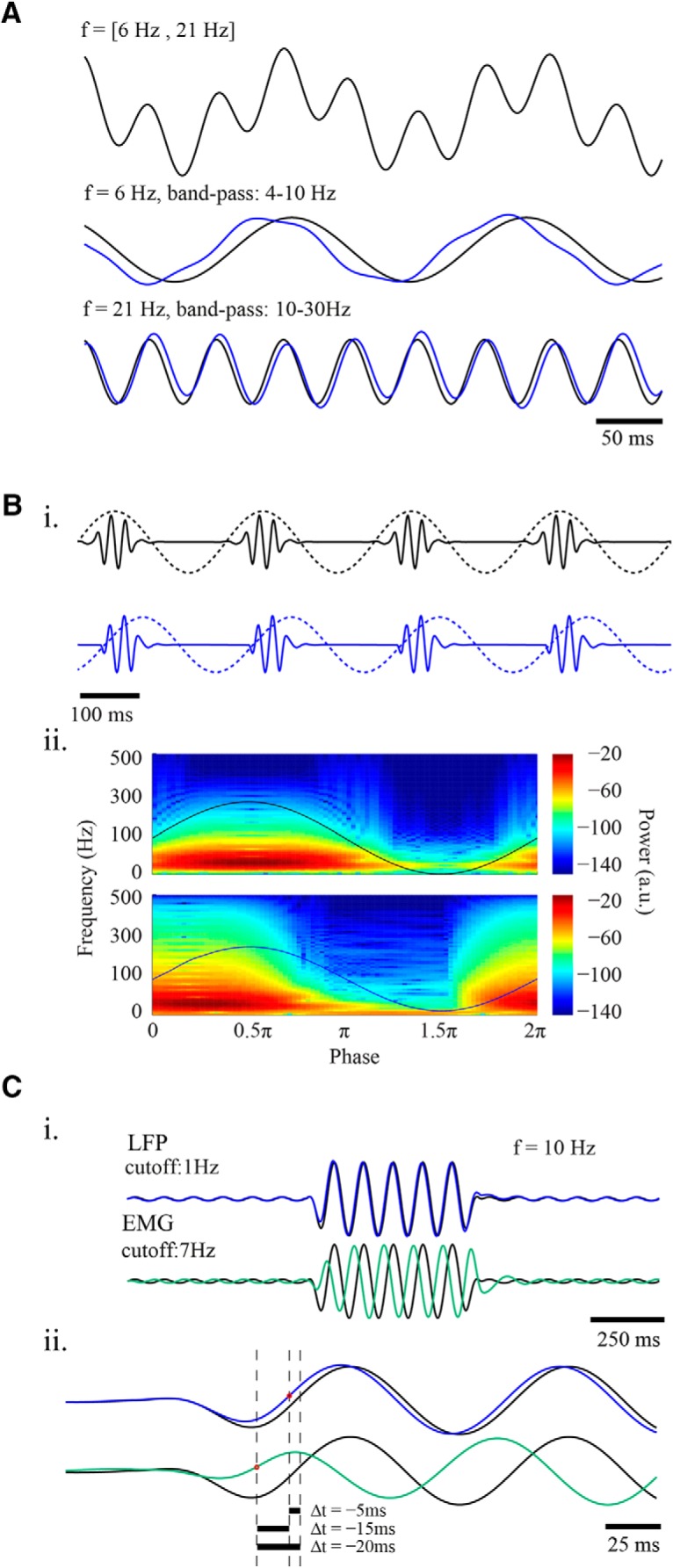
Differential phase shifts of different frequencies. Phase changes induced by a high-pass four-pole Butterworth filter in different examples (black, raw signal; blue, filtered signal). ***A***, Time shifts induced by narrow band filters in the θ (top) and β (bottom) bands, overlaid on the original oscillations constituting the signal. ***B***, Effects of secondary filtration on coupling of θ and γ band oscillations. Traces (i) of coupled θ (4 Hz) and γ (40 Hz) band oscillations, before (top) and after (bottom) filtration (3–20 Hz and 30–80 Hz two-poles Butterworth filters, respectively). Spectrograms (ii) of the γ band frequency phase locked to the θ wave, before (top) and after (bottom) filtration. ***C***, The effects of different filters on identical signals originating from different sources (i): LFP (top) and EMG (bottom; cutoff frequencies: 1 Hz, filtered LFP signal, blue; 7 Hz, filtered EMG signal, green); (ii) dashed black vertical lines mark the initiation of the oscillatory event, identified by threshold (mean ± SD of noise) crossing (right, raw; middle and left, 1- and 7-Hz high-pass-filtered signal, respectively).

The temporal disruption of different oscillations within the same signal may also occur in cases in which the oscillatory frequencies are close to each other. One typical example can take place after the extraction via filtering of narrow oscillation bands such as the θ (4–10 Hz) and β (10–30 Hz) bands (Buzsáki and Draguhn, 2004). In these cases, the temporal distortion may be exacerbated by the secondary filters applied to the wide-band filtered signal. The different filters used for each band serve to separate the frequencies of interest from the wide-band signal but cause a frequency-dependent phase distortion that disrupts both the internal timing within each narrow band signal as well as the relationship between the different narrow band signals (Fig. 3*A*). Analyses aimed at uncovering the interaction between two oscillations bands such as cross-frequency measures suffer from increased effects of phase distortions. A common example for this situation is the commonly studied coupling between θ and γ band oscillations (Tort et al., 2008). The secondary filtration of the signal using different filters, for extraction of the two bands, may lead to a further distortion of the phase-locking and temporal relationship between the two frequency bands (Fig. 3*B*).

The effects of filter-based phase shifts are compounded when multiple signals from different sources are compared. A common practice in neuroscience is to compare oscillations in the neurophysiological signal with those arising from another source such as changes in the sensory input or motor output (Levy et al., 2000). Typically, the different signals are filtered using different online filters, a process which is frequently augmented by secondary offline filters. These different filters, although not affecting the magnitude of the analyzed frequencies, leads to varying changes in their phase (Fig. 3*C*). As a result, misidentification of the preceding signal and the relationship between them may occur, leading to erroneous conclusions to questions such as whether the LFP oscillations in the basal ganglia precede the hand tremor, thus potentially driving them, or whether they follow the tremor, thus representing its somatosensory reflection.

**Figure 4.**
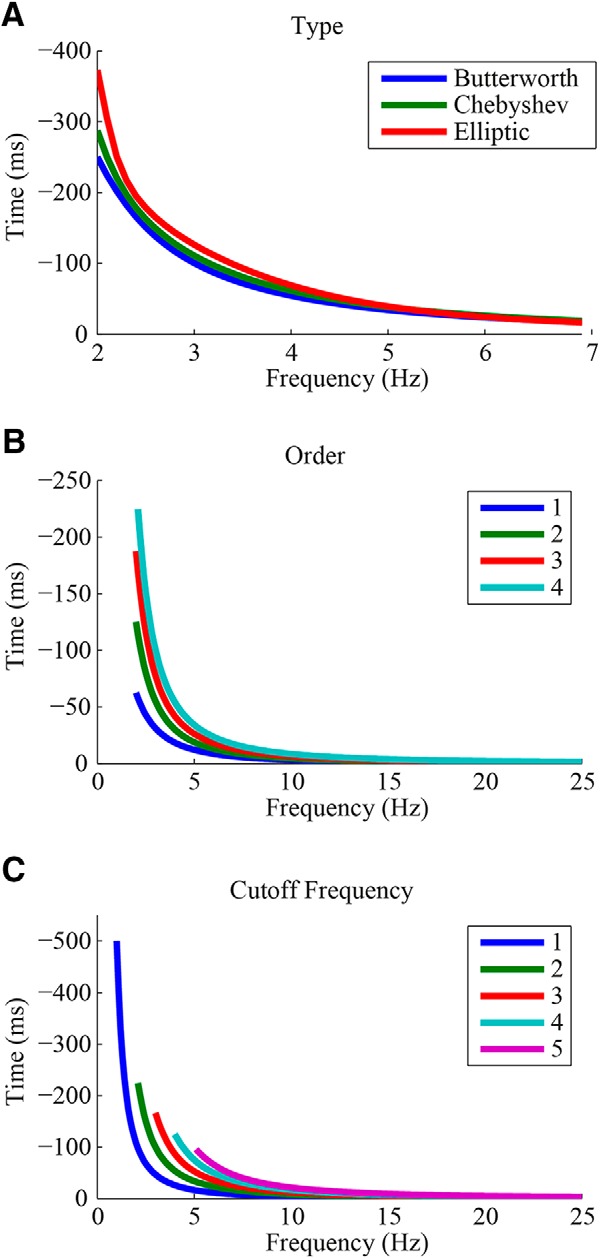
Effects of filter design on time shifts. The effects of (***A***) the filter type (Butterworth, Chebyshev and elliptic filters), (***B***) the filter order (one to four poles), (***C***) and the cutoff frequency (1–5 Hz) on filter induced time shifts.

The distortion of timing information varies across filters, depending on their specific properties. Among the properties affecting the phase response of the filter are the filter type, order, and passband frequencies. As different types of filters (e.g., Butterworth, Chebyshev, and Elliptic) differ in their amplitude responses, they also vary in their effect on phases, even for equivalent bandpass frequencies (Fig. 4*A*). Using the same filter type with the same bandpass frequencies, but with different filter orders leads to different phase responses where time shifts typically increase with the filter order (Fig. 4*B*). Changes in the cutoff frequency of the filter also lead to a change in its phase response where the time shifts increase with proximity to the cutoff frequency (Fig. 4*C*).

**Figure 5.**
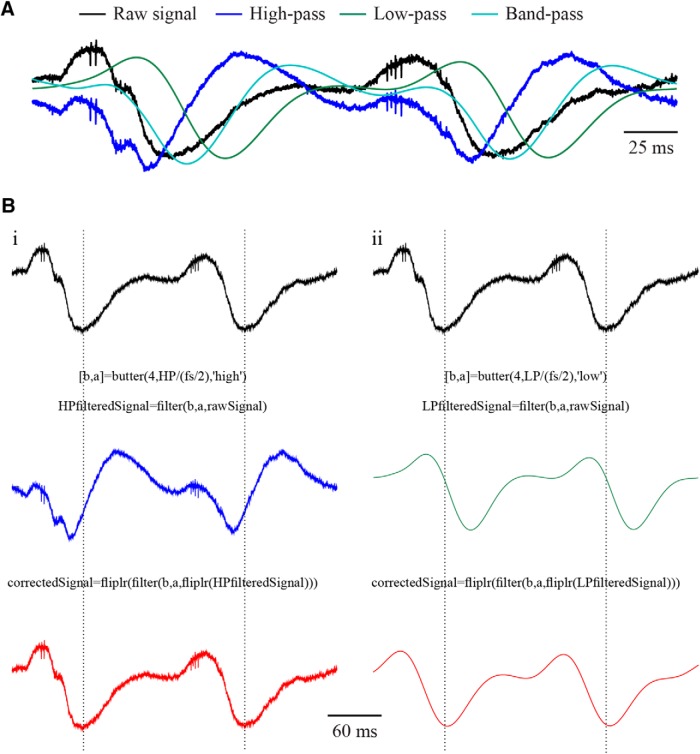
Effects of different filter designs and phase correction of an extracellularly recorded electrophysiological signal. ***A***, The effects of high (blue, cutoff frequency: 4 Hz), low (green, cutoff frequency: 20 Hz), and band (cyan, pass-band: 4–20 Hz) pass four-poles Butterworth filters on an extracellular signal recorded from a rat striatum (black). ***B***, Phase correction (red) by refiltering of the reversed filtered (i) high-pass and (ii) low-pass signals using similar filter designs.

The filter design affects the directionality of the induced phase shift such that high-pass filters produce a positive phase shift resulting in the lead of the output in relation to the input, whereas low-pass filters produce negative phase shifts resulting in delayed output, and bandpass filters induce a combination of both positive and negative phase shifts (Hartmann, 1998; Jacob, 2004; Eggleston, 2011; Fig. 5*A*). The directionality of the phase shift is derived from the electrical properties of the filter in a case of hardware-based filtering, or by the mathematical definitions of it, in a case of a software-based filtering, and is independent with the causality of the filter. These properties, and others, aggregate to exacerbate the distortions when signals are compared across different studies and/or labs, in particular since most neurophysiological manuscripts do not explicitly describe the full set of filter properties used both offline and online, rendering their comparison problematic.

## Correcting for Phase Shifts

The extent of the filter-based phase shifts and the temporal lags derived from them can be evaluated by the filter’s phase response. LP filters cause a constant time delay in all frequencies while maintaining the temporal structure of the signal. The more common NLP filters lead to differential time shifts across frequencies causing both a change in the timing of individual components within the signal and a distortion of their temporal composition. Zero-phase (ZP) filters, in which the phase shifts of all frequencies are zero, preserve the temporal properties of the signal. ZP filters, however, are not applicable in online applications. Given the exact properties of the filters applied online, the original timing of the signal can be restored, mimicking the function of a ZP filter. In an offline correction process, a filter, similar in its properties to the online filter, is applied on the reversed signal, leading to a shift of the phases back to zero, restoring the timing of the distorted signal (Longini et al., 1975; Yael and Bar-gad, 2017; Fig. 5*B*). Due to the impact of the filter design on its phase response, this process can only be achieved when the specific properties of the filter are known. Thus, while the correction for the distortions generated by filters implemented by the researcher, typically in software, is straightforward, the correction process for ready-made filters received from external sources, in both hardware and software, is typically harder, as these filters are encapsulated and their specification are in many cases obscure. Additionally, it should be recalled that residual phase distortions, such as those resulting from the properties of the electrodes and downstream parts of the electronic circuits also contribute to the deviation of the recorded signal from the real one (Magistretti et al., 1998; Nelson et al., 2008; Nelson and Pouget, 2010; Tanner et al., 2015). These factors in many cases are not explicitly known by the experimenter and are thus typically harder to compensate for.

## Conclusion

Filter-induced phase shifts can potentially impact the majority of electrophysiological signals, starting as early as in the initial stages of data acquisition. Multiple research fields in neuroscience deal with oscillatory signals, including epilepsy (Worrell et al., 2004), Parkinson’s disease (Silberstein et al., 2003), sleep (Steriade et al., 1993), memory (Klimesch, 1999), learning (Caplan et al., 2003), motor activity (Sanes and Donoghue, 1993), etc. These studies, as well as those focusing on the exact timing of components of neuronal activity (Miwakeichi et al., 2004) or cross-frequency coupling of neuronal oscillations (Tort et al., 2008) may suffer from the induced temporal distortion of their studied signals.

While multiple studies deal with the issue of filter induced changes of waveforms and amplitudes within electrophysiological signals (Bénar et al., 2010; Acunzo et al., 2012; Widmann and Schröger, 2012; Tanner et al., 2016), this manuscript discusses the impact of filters on timing information within filtered signals. The filtering process changes the phases of oscillations within the signal, leading to time delays that are either constant across frequencies in the case of LP filters, or vary as a function of frequency in the typical case of NLP filters. In the case of NLP filters, frequencies closer to the cutoff frequency of the filter are shifted to a larger extent than remote frequencies, resulting in a disruption of the internal order within the signal. In the case of LP filters, the internal composition of the signal is preserved, but its relative timing is shifted. In contrast to the effect of filters on the amplitudes of the signal, their considerable effect on the phase is usually overlooked. These effects are crucial to studies on the temporal properties of signals involving causality, the function of neuronal networks, time series, and multiple other time-based and wave form-based analyses. These effects generate a signal which is commonly considered to be the equivalent of the raw signal, but in fact comprises distorted phases. This problem is compounded when the signal is separated into its constituent frequencies by a secondary filtering or when two separate signals undergoing different filtering processes are compared. Since ZP filtering is not applicable online, these phase shifts are present in all recorded electrophysiological signals. Given the specific properties of the filters applied to the signal, this crucial effect can however be offline reversed and the distortion corrected. Currently, this is a major caveat of scientific reports as the full details of the filters used in all the stages of the data processing are typically missing or obscure. A full description of the filter’s properties within manuscripts will allow an independent evaluation of the extant of time shifts and will enable the comparison between studies performed using different filters.

## Synthesis

Reviewing Editor: Christophe Bernard, INSERM & Institut de Neurosciences des Systèmes Decisions are customarily a result of the Reviewing Editor and the peer reviewers coming together and discussing their recommendations until a consensus is reached. When revisions are invited, a fact-based synthesis statement explaining their decision and outlining what is needed to prepare a revision will be listed below. The following reviewer(s) agreed to reveal their identity: William Stacey, Anton Ivanov

Dear authors,

We have discussed extensively your paper. The reviewers and myself agree that the story is potentially very important, and potentially a must read for all scientists using electrophysiological recordings. Because the issue may be critical, for example it questions all works on cross-frequency coupling. Therefore, such a paper should be rock-solid. Our goal here is to try help you, since we are all very supportive of the concept. However, please note that you must address the two major issues listed hereafter for the ms to go further.

We acknowledge your effort in making the concept accessible to non-engineers and scientists not versed in signal processing. However, this has a major drawback. All of us have engineering and signal processing background, and we don't know whether some incorrect statements stem from the necessary simplification, or from a lack of background knowledge from your part (no criticisms meant). Perhaps you may be able to fix it, or we could help you out. In any case, the paper should contain an engineering part that is solid (a part that could be skipped by a lay audience).

Example of a problem: Fig 1 introduces the authors concept of a negative time delay, which makes it appear that the filter would show a 3 hz wave 100 ms before it even happens. This idea is pervasive in the paper. This is really an artifact of using pure sinusoids: in a perfect sinusoid it is possible to say something with a phase shift that creates a time delay-but is it +100 or -160? Did the wave shift to the side in time, or did each amplitude of each point just shift in phase? With a perfect sine it is the same thing, but for a physiological signal such is not the case. And it is clearly not possible for a real-time filter to display a wave 100 ms before it occurs. But throughout the paper, it is implied that filtering will make signals appear before they actually occur. It is clearly easier to talk about phase delays if one transforms them into time shifts, but they really are not the same thing. This whole description is thus an overly-simplistic treatment of digital signal processing, using somewhat-true examples to make the concept approachable to a non-technical reader. But to a technical reader, it is disturbing to read. This is not robust signal processing.

The question of whether there are filters that can actually make a signal appear early must be answered. Certainly, no FIR filter (or other causal) filter can do this, but there are non-causal filters that could do so. Causal and non-causal filters need to be discussed in this paper if the authors wish to talk about true negative time shifts). After clarifying when they truly think the signal has shifted negatively in time, the authors should address what situations might produce that. Obviously, this can only happen in post processing, which must be clearly explained. It is our opinion that it will be hard to conceive of a traditional filter that would do this. Of note, the authors make a point of saying that a zero-phase filter is not available on-line, they should also note that it is also impossible to have a non-causal filter on-line.

The other feature to fix is the physiological relevance. We all agree that you need to real examples of those effects. For instance, sample tracings with different frequency components could be passed through different filters and analyzed, showing the effect of these differences. Could they, for instance, show that theta/gamma or slow oscillations/sharp wave ripple coupling are actually dependent upon the specific filter? For example, you could digitize a real signal where cross-frequency coupling occurs, make it analog again and feed it to the amplifier. Alternatively, you can generate a physiological-like signal. You could also use simultaneous DC/AC recordings, and demonstrate what AC is doing to the phase. You need to help to the reader to identify when these effects happen with real examples. It would also be important to show how to fix the problem.

In conclusion, it is essential to address both issues : soundness in the engineering part and showing that phase shift indeed happens in real signals (apart from those reported in your PloSone paper).

Scholarship: There are several areas of past work not addressed by the authors. For instance, the real-signal analysis described above has been done before in the EEG field. Several papers have looked at how filters can distort EEG, affect high frequency oscillations and produce artifacts. Those papers typically show the effects with real signals. There are several groups who have looked at the filtering properties of electrodes of different materials, different tissues, and different acquisition equipment. Finally, there is a vast amount of literature of these effects within Digital signal processing.

You may want to check:

Oppenheim A V., Schafer RW, Buck JR, Schafer Ronald W. BJROA V. Discrete Time Signal Processing. 2nd editio. Book. Upper Saddle River, New Jersey 07458: Prentice-Hall, Inc.; 1999. 870 p.

Nelson MJ, Pouget P, Nilsen E a, Patten CD, Schall JD. Review of signal distortion through metal micro-electrode recording circuits and filters. J Neurosci Methods. 2008

http://www.analog.com/en/analog-dialogue/articles/phase-relations-in-active-filters.html

You should mention that filters are often under the hood-not noticed by the user, and often poorly understood. In addition, it is often difficult to find out the details of the filters from the manufacturers. But when data is available, a table showing which type of filter (manufacturer-specific?) is doing what would be very useful to everyone.

## Author Response

Dear authors,

We have discussed extensively your paper. The reviewers and myself agree that the story is potentially very important, and potentially a must read for all scientists using electrophysiological recordings. Because the issue may be critical, for example it questions all works on cross-frequency coupling. Therefore, such a paper should be rock-solid. Our goal here is to try help you, since we are all very supportive of the concept. However, please note that you must address the two major issues listed hereafter for the ms to go further.

We acknowledge your effort in making the concept accessible to non-engineers and scientists not versed in signal processing. However, this has a major drawback. All of us have engineering and signal processing background, and we don't know whether some incorrect statements stem from the necessary simplification, or from a lack of background knowledge from your part (no criticisms meant). Perhaps you may be able to fix it, or we could help you out. In any case, the paper should contain an engineering part that is solid (a part that could be skipped by a lay audience).

Filter based phase distortion has been known to engineers for a long time and we do not claim to have anything to renew to them. In this manuscript we aim only to present the problem of filter-based distortion of timing information within electrophysiological signals to experimentalists with a limited background in engineering, and to bring very simple common examples of this phenomenon. This phenomenon, however, is not unique to neurophysiology, and thus, there are multiple well-written sources of professional literature dealing with it at all levels ranging from the amateur to the extremely sophisticated. We added citations of such resources to the revised manuscript in the relevant places for further reading. Regarding your concerns regarding incorrect statements, we will gladly work with the reviewers to address specific issues.

As we (DY & IBG) are brain scientists and not engineers, to facilitate the engineering approach to the manuscript we collaborated with an EE in the revision who was added as a co-author (JJV) on the revised manuscript.

Example of a problem: Fig 1 introduces the authors concept of a negative time delay, which makes it appear that the filter would show a 3 hz wave 100 ms before it even happens. This idea is pervasive in the paper. This is really an artifact of using pure sinusoids: in a perfect sinusoid it is possible to say something with a phase shift that creates a time delay-but is it +100 or -160? Did the wave shift to the side in time, or did each amplitude of each point just shift in phase? With a perfect sine it is the same thing, but for a physiological signal such is not the case. And it is clearly not possible for a real-time filter to display a wave 100 ms before it occurs. But throughout the paper, it is implied that filtering will make signals appear before they actually occur. It is clearly easier to talk about phase delays if one transforms them into time shifts, but they really are not the same thing. This whole description is thus an overly-simplistic treatment of digital signal processing, using somewhat-true examples to make the concept approachable to a non-technical reader. But to a technical reader, it is disturbing to read. This is not robust signal processing.

The question of whether there are filters that can actually make a signal appear early must be answered. Certainly, no FIR filter (or other causal) filter can do this, but there are non-causal filters that could do so. Causal and non-causal filters need to be discussed in this paper if the authors wish to talk about true negative time shifts). After clarifying when they truly think the signal has shifted negatively in time, the authors should address what situations might produce that. Obviously, this can only happen in post processing, which must be clearly explained. It is our opinion that it will be hard to conceive of a traditional filter that would do this. Of note, the authors make a point of saying that a zero-phase filter is not available on-line, they should also note that it is also impossible to have a non-causal filter on-line.

Although negative time delays, and specifically the ones created by filters which do not use future inputs (i.e. causal filters), stand in sharp contrast to common sense - they do exist. However, their interpretation as predicting the future should be taken with care (see nice column by Andor Bariska: Time Machine, Anyone?). This phenomenon occurs in the case of sinusoidal functions, as brought for simplicity in the original manuscript, but also exists in cases of more complex non-periodic signals such as experimentally recorded electrophysiological signals shown in the revised manuscript (new Figure 4) and simulated band-limited noise shown below (Fig. 1). This phenomenon is explained and discussed in multiple resources of engineering literature, in cases of hardware and software based filtration. This can be extracted mathematically from the formulas describing the filters (for example: Eggleston, 2011) or intuitively from other descriptions, see for example links to intuitive explanations in the levels of hardware-based and software-based filtering.

Theoretically, of course, a system in which the output predicts or leads the input may only be a non-causal one. However, negative time delays occur in high-pass filters independently of the causality of the filter. As explained above, the positive phase delays which are translated into negative time delays are created by the design (the mathematical definitions) of the filter (in the case of software-based filtration) or by the electrical properties of it (in the case of hardware-based filtration). A simple example demonstrating this phenomenon is a differentiator (which can be applied as a high pass filter in both hardware and software). In this case - the output of the filter is simply the first derivative of the input. The derivative leads the original function; however, it does not use future inputs. This can also be demonstrated by a causal 2-poles high-pass Butterworth filter, in which the output is dependent only on present and past samples of the output and input (equation 1). Yet, the output of the signal leads its input (Fig.1).

y(n)=‵_(i=0)^2′(b_i*x(n-i)) +‵_(j=1)^2′(a_i*y(n-i))

=b_2*x(n-2)+b_1*x(n-1)+b_0*x(n)+a_2*y(n-2)+a_1*y(n-1)

b_0=1,b_1=-2,b_2=1,a_1=1.9982228473,a_2=-0.9982244250

Equation 1: 2-poles high-pass Butterworth filter recurrence relation, cutoff frequency: 2 Hz, Fs = 10000Hz.

Figure 1: Temporal effect of Butterworth filter. Signals following 2-poles high-pass Butterworth filter (cutoff frequency: 2 Hz, fs = 10000 Hz). (A) 5 Hz sinusoidal function,(B): limited-band (2-10 Hz) noise, (C) 5 Hz sinusoidal step function. Signals were filtered using the direct implementation of the Butterworth filter shown in Equation 1 (red) and by the generic Matlab code (dashed blue) (both functions attached in the appendix).

The other feature to fix is the physiological relevance. We all agree that you need to real examples of those effects. For instance, sample tracings with different frequency components could be passed through different filters and analyzed, showing the effect of these differences. Could they, for instance, show that theta/gamma or slow oscillations/sharp wave ripple coupling are actually dependent upon the specific filter? For example, you could digitize a real signal where cross-frequency coupling occurs, make it analog again and feed it to the amplifier. Alternatively, you can generate a physiological-like signal. You could also use simultaneous DC/AC recordings, and demonstrate what AC is doing to the phase. You need to help to the reader to identify when these effects happen with real examples. It would also be important to show how to fix the problem.

A new figure summarizing multiple issues mentioned in the manuscript and demonstrating them on real data was added to the manuscript (Fig. 4A). In this figure we demonstrate the effect of different (high/low/band pass) filters on a physiological extracellularly recorded signal to its different constituting frequencies. In addition, we demonstrate the correction of shifted phases of these signals (Fig. 4B). We also added references which deal specifically with the process of phase correction and signal reconstruction.

In conclusion, it is essential to address both issues : soundness in the engineering part and showing that phase shift indeed happens in real signals (apart from those reported in your PloSone paper).

Scholarship: There are several areas of past work not addressed by the authors. For instance, the real-signal analysis described above has been done before in the EEG field. Several papers have looked at how filters can distort EEG, affect high frequency oscillations and produce artifacts. Those papers typically show the effects with real signals. There are several groups who have looked at the filtering properties of electrodes of different materials, different tissues, and different acquisition equipment. Finally, there is a vast amount of literature of these effects within Digital signal processing.

You may want to check:

Oppenheim A V., Schafer RW, Buck JR, Schafer Ronald W. BJROA V. Discrete Time Signal Processing. 2nd editio. Book. Upper Saddle River, New Jersey 07458: Prentice-Hall, Inc.; 1999. 870 p.

Nelson MJ, Pouget P, Nilsen E a, Patten CD, Schall JD. Review of signal distortion through metal micro-electrode recording circuits and filters. J Neurosci Methods. 2008

http://www.analog.com/en/analog-dialogue/articles/phase-relations-in-active-filters.html

There are multiple studies dealing with the effect of filtration on signals. However, most of these studies deals with the issue of the amplitudes of different frequencies rather than their phases and the derived timing. We added multiple references, amongst them some of these studies, to the revised manuscript. Nelson 2008 was already cited in the original manuscript and Oppenheim 1999 was added together with other engineering-centric literature resources which were added to the revised manuscript.

You should mention that filters are often under the hood-not noticed by the user, and often poorly understood. In addition, it is often difficult to find out the details of the filters from the manufacturers. But when data is available, a table showing which type of filter (manufacturer-specific?) is doing what would be very useful to everyone.

The issue of missing details in filters' description given by both manufacturers and researchers is indeed an important matter. This missing information hinders the evaluation of the phase shifts induced by the filtration process and the comparison between different studies performed in different laboratories. We refer this issue along the manuscript in different contexts. To emphasize the importance of a full description of these details, we added a specific mention of this point in the conclusion of the revised manuscript.

References

Eggleston D (2011) Basic Electronics for Scientists and Engineers. Cambridge University Press.

function butterworthImplementation ()

% 2-poles high-pass Butterworth filter, fs=10000Hz

% Recurrence relation was extracted from

% http://www-users.cs.york.ac.uk/~fisher/mkfilter/trad.html

%COMPUTE INPUT X1 - sinusoidal function

fs=10000; %Hz

T=10;%s

freq=5;%Hz

t=[0:T*fs-1]/fs;

x=sin(2*pi*t*freq);

figure;

subplot (3,1,1); hold on;

computeY (x,fs);

title ([num2str(freq) “ Hz Sin. Func. following 2-poles High-pass Butterworth filter (2Hz cutoff freq.)” ])

xlim([3 5]);

ylim([-1.2 1.2]);

%COMPUTE INPUT X2- noise

noise=randn(1,T*fs);

BP=[2 10];

[bbp,abp]=butter(2,BP/(fs/2),‘bandpass’);

noise=filtfilt(bbp,abp,noise);

subplot (3,1,2); hold on;

computeY (noise,fs);

title ([‘Noise (’ num2str(BP(1)) ‘-’ num2str(BP(2)) “Hz) following 2-pole High-Pass Butterworth filter (cutoff freq.: 2Hz )” ])

ylim ([-0.15 0.15]);

xlim([3 5]);

% COMPUTE INPUT X3 -

fs=10000; %Hz

T=1;%s

freq=5;%Hz

t=[0:T*fs-1]/fs;

x=sin(2*pi*t*freq);

x= [zeros(1,2*length(x)) x zeros(1,2*length(x))];

subplot (3,1,3); hold on;

computeY (x,fs);

title ([num2str(freq) “ Hz Sin. Step Func. following 2-poles High-pass Butterworth filter (2Hz cutoff freq.)” ])

xlim([1.5 3.5]);

ylim([-1.2 1.2]);

function []=computeY (x,fs)

%COMPUTE OUTPUT Y

y=zeros(1,length(x));

for i=3:(length(x))

y(i)=(1*x(i-2))+(-2*x(i-1))+(1*x(i))+(-0.9982244250*y(i-2))+(1.9982228473*y(i-1));

end

%compare to matlab's Butterworth

[b,a]=butter(2, 2/(fs/2), ‘high’);

yMatlab=filter(b,a,x);

% plot

plot ([0:length(x)-1]/fs,x,‘k’,‘linewidth’,2);

plot([0:length(y)-1]/fs,y,‘r’,‘linewidth’,2);

plot ([0:length(yMatlab)-1]/fs,yMatlab,‘b:’,‘linewidth’,2);

legend ({‘Original’, ‘Manual’, ‘MATLAB’}, ‘location’, ‘northeastoutside’);

xlabel (‘Time (s)’);
